# Disruption of HPV16-E7 by CRISPR/Cas System Induces Apoptosis and Growth Inhibition in HPV16 Positive Human Cervical Cancer Cells

**DOI:** 10.1155/2014/612823

**Published:** 2014-07-20

**Authors:** Zheng Hu, Lan Yu, Da Zhu, Wencheng Ding, Xiaoli Wang, Changlin Zhang, Liming Wang, Xiaohui Jiang, Hui Shen, Dan He, Kezhen Li, Ling Xi, Ding Ma, Hui Wang

**Affiliations:** ^1^Department of Obstetrics and Gynecology, Tongji Hospital, Tongji Medical College, Huazhong University of Science and Technology, Wuhan, Hubei 430030, China; ^2^Cancer Biology Research Center, Tongji Hospital, Tongji Medical College, Huazhong University of Science and Technology, Wuhan, Hubei 430030, China; ^3^Department of Neurology, Tongji Hospital, Tongji Medical College, Huazhong University of Science and Technology, Wuhan, Hubei 430030, China

## Abstract

High-risk human papillomavirus (HR-HPV) has been recognized as a major causative agent for cervical cancer. Upon HPV infection, early genes E6 and E7 play important roles in maintaining malignant phenotype of cervical cancer cells. By using clustered regularly interspaced short palindromic repeats- (CRISPR-) associated protein system (CRISPR/Cas system), a widely used genome editing tool in many organisms, to target HPV16-E7 DNA in HPV positive cell lines, we showed for the first time that the HPV16-E7 single-guide RNA (sgRNA) guided CRISPR/Cas system could disrupt HPV16-E7 DNA at specific sites, inducing apoptosis and growth inhibition in HPV positive SiHa and Caski cells, but not in HPV negative C33A and HEK293 cells. Moreover, disruption of E7 DNA directly leads to downregulation of E7 protein and upregulation of tumor suppressor protein pRb. Therefore, our results suggest that HPV16-E7 gRNA guided CRISPR/Cas system might be used as a therapeutic strategy for the treatment of cervical cancer.

## 1. Introduction

Cervical cancer is the second most common cancer in women worldwide [1]. High-risk human papillomavirus (HR-HPVs), especially HPV16 and HPV18, is considered major causative agent for cervical cancer [2]. Oncogenes E6 and E7 are expressed in the early stage of HPV infection, and their functions are to disrupt normal cell cycle and to maintain a transformed malignant phenotype [3, 4]. For instance, E7 protein binds to cullin 2 ubiquitin ligase complex and leads to the ubiquitination and degradation of the retinoblastoma (pRb) tumor suppressor [5]. And in the absence of pRb, the E2F family of transcription factors is released and host cell proliferation is promoted [5]. Therefore, they are attractive targets for cancer gene therapy. 

**Figure 1:**
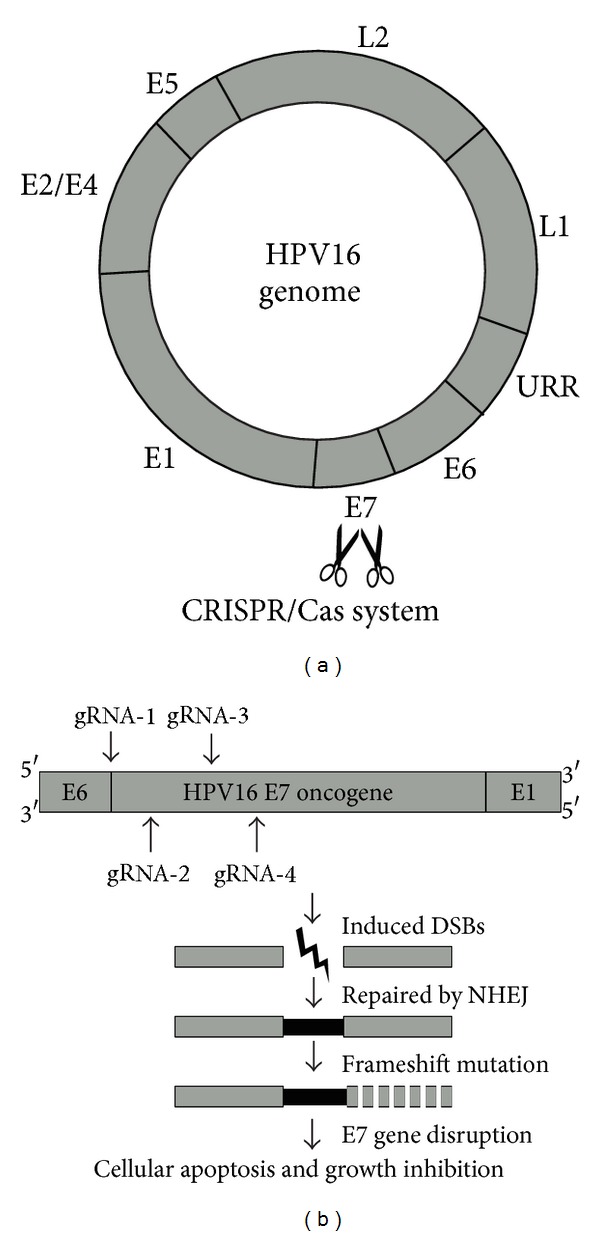
Schematic representation of HPV16-E7 gene editing using the CRISPR/Cas system. (a) The CRISPR/Cas system-mediated E7 gene targeting of HPV16 genome. (b) Schematic representation of the four customized gRNAs disrupting the E7 gene. The CRISPR/Cas system could induce double strand breaks of the E7 oncogene, which lead to NHEJ repair and frameshift mutation. Disruption of the E7 gene would further result in apoptosis and growth inhibition of HPV16-positive cells. Black arrows represent Cas9 enzyme-mediated DSB breaking sites upon gRNA recognition.

The CRISPR/Cas system is a newly developed programmable RNA-guided endonuclease system. And it has emerged as a powerful genome editing tool in many organisms including prokaryotes,* C. elegans,* and zebrafish [6–8]. Consisting of a site-specific single-guide-RNA (sgRNA) and a Cas9 enzyme, the system can basically target any genomic site in the form of 5′-N20NGG-3′ [9]. Upon recognition at a determined genomic site complementary to sgRNA sequence, Cas9 enzyme induces double strand breaks (DSBs) (Figure 1). DSBs are mainly repaired through the mutagenic nonhomologous end joining (NHEJ) repair pathway, leading to disruption of the targeted gene [10].

In this study, we used the CRISPR/Cas system to cleave the E7 oncogene in HPV16 positive cervical cancer cell lines. We showed that mutations induced by the CRISPR/Cas directly lead to apoptosis and growth inhibition in HPV16-positive cells, but not in HPV16-negative cells. Disruption of E7 gene and subsequent loss of E7 oncoprotein restored the expression of tumor suppressor pRb. Our data indicated that HPV16-E7 gRNA-guided CRISPR/Cas is a potential therapeutic strategy for treatment of cervical cancer.

## 2. Materials and Methods

### 2.1. Cell Culture and Transfection

SiHa, Caski, C33A, and HEK293 cell lines were purchased from ATCC (American Type Culture Collection) and cultured in Dulbecco's modified Eagle's medium (Sigma) supplemented with 10% FBS (Gibco), 100 U/mL penicillin, and 100 *μ*g/mL streptomycin (Invitrogen). The cells were transfected at 80% confluency. We used X-tremeGENE HP DNA transfection reagent (Roche) for SiHa, C33A, and HEK293 cells and jetPEI polymer-based DNA transfection reagent (Polyplus) for Caski cells following the manufacturer's protocol. The total amount of DNA added to each well in 24-well plates and 6-well plates was 1 *μ*g and 2 *μ*g, respectively. Ratio of the reagent to DNA in each well was optimized preexperimentally. The experiments were repeated twice.

### 2.2. Plasmids

We designed four gRNAs targeting HPV16-E7 gene in our lab following the protocol of Mali et al. [9] and synthesized them in Genewiz Company (China). Since HPV16E6-gRNA-1 was designed targeting HPV16-E6 gene and was proved to be ineffective preexperimentally, it was applied as a negative control. Sequences of the customized gRNAs used in this study were described in Table 1. The plasmid encoding Cas9 was kindly obtained from Addgene.

### 2.3. Single-Strand Annealing Assay

**Figure 2:**
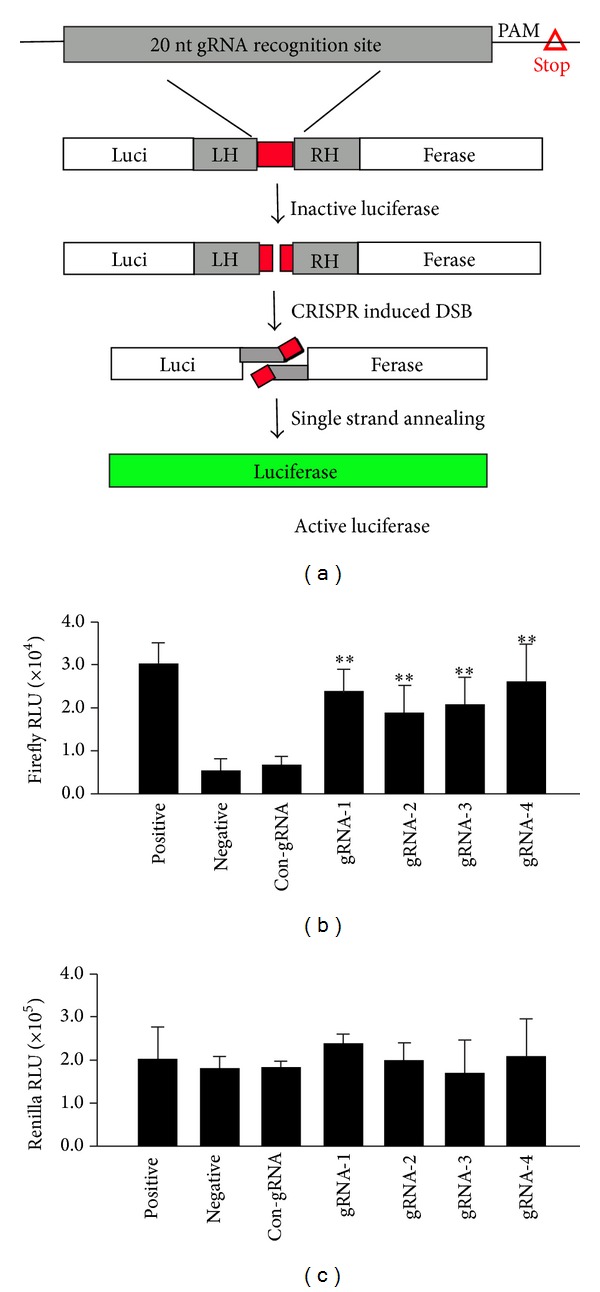
Evaluation of gRNA/Cas9-mediated HPV16-E7 DNA cleavage by the SSA assay. (a) Schematic representation of luciferase assay. Inactive luciferase gene is separated by left homology arm (LH), 20 nt gRNA recognition site, corresponding PAM sequence, a stop codon, and right homology arm (RH). When the CRISPR gRNA/Cas9 system causes DSBs at specific recognition sites, an active luciferase will form due to SSA repair pathway. Cas9 plasmid, site-specific gRNA, pSSA Rep-gRNA, and renilla plasmid were cotransfected into HEK293 cells. At 48 h after transfection, the firefly luciferase (b) and renilla luciferase (c) activity were measured by a microplate reader. Cells transfected with GZF3-L3, GZF1-R3, and pSSA Rep3-1 plasmids were used as positive control and cells treated with only Cas9 plasmid were applied as negative control. Cells treated with Con-gRNA (HPV16E6-gRNA-1) which had been proved to be inactive preexperimentally and Cas9 enzyme together were used as a control. RLU represents relative light units (**P < 0.01 compared to the negative test, *n* = 6, per Student's *t*-test).

Construction of the SSA luciferase reporter pSSA Rep3-1 plasmid has been described previously [11]. The pSSA Rep3-1 and control GZF3-L3 + GZF1-R3 ZFN plasmids were kindly provided by Professor David Segal. Briefly, a CRISPR sgRNA target sequence, its corresponding PAM sequence, and a stop codon were inserted into the direct repeat halves of the firefly luciferase gene pSSA Rep3-1 (Figure 2(a)), which was named as pSSA Rep-gRNA. 400 ng of each Cas9 plasmid, 100 ng of gRNA plasmid, 100 ng of pSSA Rep-gRNA, and 25 ng of pRL-TK-Renilla luciferase (Promega) were cotransfected into HEK293 cells in 24-well plates. At 48 h after transfection, firefly luciferase and Renilla activities were determined according to the protocol of the Dual-Luciferase Reporter Assay System (Promega). Luciferase activity was monitored with a microplate luminometer (BioTek). All the experiments were repeated three times.

### 2.4. Flow Cytometry Detection of Apoptosis

The cells were cotransfected with 0.8 *μ*g of Cas9 plasmid and 0.2 *μ*g of gRNA plasmid in 24-well plates. At 48 h after transfection, they were collected and double stained with fluorescein isothiocyanate- (FITC-) conjugated annexin V (annexin V-FITC) and propidium iodide (PI) using an Annexin V-FITC Apoptosis detection kit (KeyGen BioTech) according to the manufacturer's instructions. Apoptosis rates of all of the four CRISPR/Cas system treated cell lines were analyzed using a FACS Calibur (BD Bioscience) to calculate the induced cell death. Data was analyzed using BD Cell Quest software.

### 2.5. CCK-8 Assay

In vitro cell proliferation was determined using Cell Counting Kit-8 (CCK-8; Beyotime). Transfected with the gRNA-4/Cas9 plasmids with 1 × 10^4^ cells/well, cells were trypsinized and seeded onto 96-well plates at 24 h after transfection. At 0 h, 24 h, 48 h, 72 h, and 96 h after being seeded onto 96-well plates, 10 *μ*L CCK-8 solution was added in each well followed by 2.5 h incubation at 37°C. We measured the absorbance values at 490 nm with a Microplate Reader (Bio-Rad, USA).

### 2.6. Isolation of Cellular DNA and T7 Endonuclease I Assay

Based on FCM data of the four gRNA/Cas9 groups, gRNA-4/Cas9 manifested the highest apoptosis rate; therefore, we chose the gRNA-4/Cas9 group for further test. Genomic DNA was extracted using DNeasy Blood & Tissue Kit (QIAGEN) according to the manufacturer's instructions after maintaining the transfected cells for 48 h. The T7 Endonuclease I (T7EI) assay was performed as previously described [12]. After PCR-amplifying the region containing the targeting sites of HPV16-E7-gRNA, the products amplified from SiHa and Caski cells were denatured by heating and reannealed to form heteroduplex DNA. Each of 200 ng PCR products was treated with 2 units of T7 Endonuclease (New England Biolabs) for 15 min at 37°C and then separated using a 10% TBE polyacrylamide gel. The primers used were listed (Table 2).

### 2.7. Western Blotting

In order to detect the effect of CRISPR/Cas system on protein level of E7 after E7 gene disruption and its subsequent effect on pRb protein, we performed Western blotting in gRNA-4/Cas9 treated SiHa and Caski cells. We seeded SiHa and Caski cells onto 6-well plates and transfected the cells with 1.6 *μ*g of Cas9 plasmid and 0.4 *μ*g of gRNA plasmid. After 48 h, cellular proteins were extracted and separated using Western blotting analysis. The final results were detected by horseradish peroxidase-conjugated anti-rabbit Ig secondary antibody using a SuperSignal West Pico kit (ThermoFisher Scientific). The primary antibodies used were anti-HPV16 E7 (orb 10573, Biorbyt), anti-pRb (10048-2-Ig, Proteintech), and anti-*β*-actin (AA128, Beyotime). *β*-actin was used as a loading control.

### 2.8. Statistical Analysis

Data was performed with SPSS 12.0 (SPSS Inc., Chicago, USA) and expressed as mean ± SD. Statistical analysis was assessed by Student's *t*-test. Statistical significance was defined as *P < 0.05.

## 3. Results

### 3.1. HPV16-E7-Specific gRNAs/Cas9 Induced DSBs

We applied a mammalian cell-based single-strand annealing (SSA) assay to investigate whether the customized site-specific gRNAs/Cas9 could disrupt the E7 gene in HEK293 cells. The DNA sequences of the four customized gRNAs and their corresponding PAM sequences were constructed into the direct repeat halves of luciferase gene. When DSBs were induced by gRNAs/Cas9, the stop codon was removed and an intact luciferase gene was formed under the direction of SSA homologous recombination (Figure 2(a)). The Renilla luciferase plasmid was used to monitor CRISPR induced cytotoxicity. As is shown in Figure 2(b), the positive control displayed the highest signal of firefly luciferase, and signals shown by the four gRNAs/Cas9 groups were more than three times compared to negative control. These data indicated that all of the four gRNAs/Cas9 groups could lead to efficient double strand breaks (DSBs) at their corresponding target sites. Con-gRNA represented cells transfected with HPV16E6-gRNA-1/Cas9, which was proved to be ineffective preexperimentally. In addition, measurement of Renilla luciferase displayed no significant change of signal (Figure 2(c)), indicating relatively low cytotoxicity of gRNAs/Cas9 used in this study for the transfected cell lines.

### 3.2. HPV16-E7-Specific gRNAs/Cas9 Induced Apoptosis in HPV16 Positive Cell Lines

**Figure 3:**
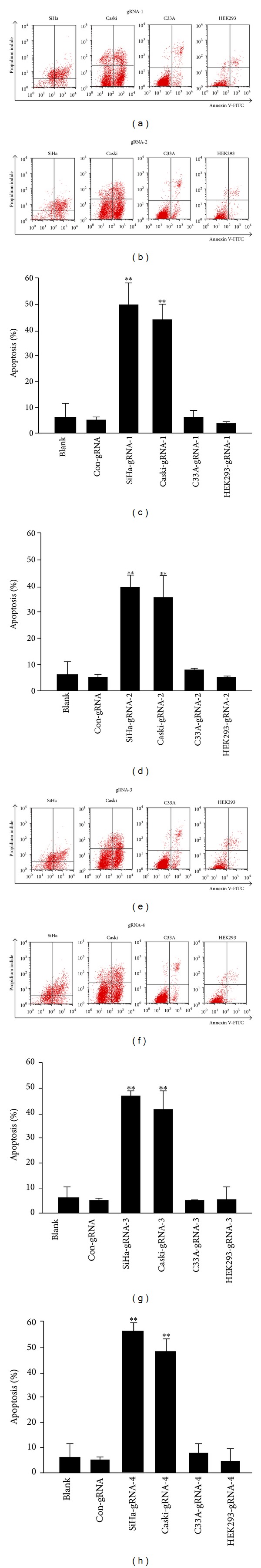
Disruption of the E7 gene could significantly enhance apoptosis monitored by FCM in SiHa and Caski cells, but not in C33A and HEK293 cells. gRNA-1/Cas9 ((a), (c)), gRNA-2/Cas9 ((b), (d)), gRNA-3/Cas9 ((e), (g)), and gRNA-4/Cas9 ((f), (h)) induced apoptosis rate in SiHa, Caski, C33A, and HEK293 cell lines (**P < 0.01 compared to blank control, per Student's *t*-test).

To determine whether the four groups of gRNAs/Cas9 could specifically induce cellular apoptosis in HPV16 positive cells, we introduced gRNAs/Cas9 plasmids into HPV16 positive SiHa and Caski cell lines, together with HPV negative C33A and HEK293 cell lines, respectively. Compared with the apoptosis rate of blank control group and con-gRNA group (apoptosis rates were less than 10%), the apoptosis rates induced by gRNA-1/Cas9, gRNA-2/Cas9, gRNA-3/Cas9, and gRNA-4/Cas9 were 50%, 40%, 47%, and 56%, respectively, in SiHa cells and 44%, 35%, 42%, and 48%, respectively, in Caski cells (Figure 3). On the other hand, apoptosis induced by the same four gRNA/Cas9 groups in HPV negative C33A and HEK293 cell lines were below 10%. Based on the data that gRNA-4/Cas9 treated groups displayed the highest rate of apoptosis both in SiHa and Caski cells, gRNA-4/Cas9 was selected for further experiments.

### 3.3. Inhibited Cellular Proliferation through E7 Disruption

**Figure 4:**
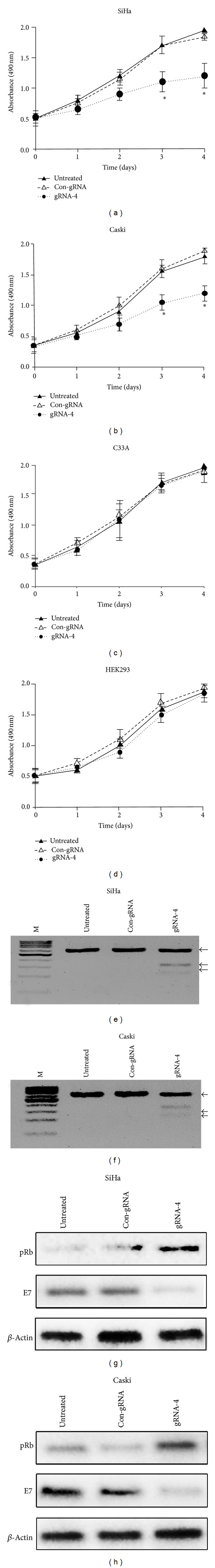
Effect of HPV16-E7 gRNA-4/Cas9 on cellular proliferation, DSB cleavage, and E7 and pRb proteins level. HPV16-E7 gRNA-4/Cas9 inhibited cellular viability in SiHa and Caski cells but not in C33A and HEK293 cells ((a)–(d)). Cellular viability of SiHa, Caski, C33A, and HEK293 cells were monitored by CCK-8 assay (*P < 0.05 compared to untreated group, per Student's *t*-test). T7E1 treatment of heteroduplex DNA in the gRNA-4/Cas9 group showed noncleaved products at 500 bp and cleaved products at 300 bp and 200 bp both in SiHa and Caski cells ((e), (f)). Black arrows indicate 500 bp, 300 bp, and 200 bp PCR products. Untreated represented cells transfected with only Cas9 plasmid and was used as a negative control. Con-gRNA meant cells treated with HPV16E6-gRNA-1/Cas9, which was proved to be inactive preexperimentally. *M* was 100 bp DNA marker. E7 protein was downregulated and pRb protein was upregulated both in SiHa and Caski cells ((g), (h)). Data were drawn from four independent experiments. *β*-Actin was used as an internal control.

We next investigated whether disruption of E7 gene could inhibit cellular proliferation in HPV16 positive SiHa and Caski cells. At 72 h and 96 h after transfection, compared to untreated groups, there was a significant reduction in cell viability in gRNA-4/Cas9 treated SiHa and Caski cells (Figures 4(a) and 4(b)). However, the absorbance rate in gRNA-4/Cas9 treated HPV16 negative C33A and HEK293 cells did not show significant difference compared to untreated groups (Figures 4(c) and 4(d)). These results indicated that gRNA-4/Cas9 could specifically inhibit the growth of HPV16 positive cervical cancer SiHa and Caski cell lines, but not HPV16 negative cervical cancer cell C33A or normal cell line HEK293.

### 3.4. CRISPR-Mediated Targeted Disruption of HPV16-E7 Gene

To assess targeted HPV16-E7 gRNA-4/Cas9-mediated disruption of E7 gene, we subjected PCR products amplified from gRNA-4/Cas9 treated SiHa and Caski cells to T7E1 assay. As is shown in Figures 4(e) and 4(f), heteroduplex DNA of the gRNA-4/Cas9 group after T7E1 treatment showed three different lengths of products at 500 bp, 200 bp, and 300 bp, respectively. The PCR products of control gRNA/Cas9 group and untreated group were not affected and could only be detected at 500 bp. Consistent with SSA assay, these results confirmed that gRNA-4/Cas9 could effectively induce DSBs at specific targeting site of E7 oncogene.

### 3.5. E7 Disruption Led to Downregulation of E7 Protein and Upregulation of pRb Protein

Western blotting analysis was performed to investigate the effect of E7 gene disruption on E7 and pRb proteins expression. Upon gRNA-4/Cas9 treatment, E7 protein level decreased more than 50% compared to untreated group, while pRb protein level was restored in both SiHa and Caski cells (Figures 4(g) and 4(h)). Protein level of the con-gRNA/Cas9 treated group in SiHa and Caski cells remained unchanged compared to untreated group. Western blotting analysis of E7 and pRb protein was not performed in HPV negative C33A and HEK293 cell lines. These data suggested that the CRISPR/Cas system targeting E7 gene could efficiently downregulate protein expression of E7 and upregulate tumor suppressor protein pRb.

## 4. Discussion

HPV oncogene E7 plays important roles in maintaining malignant phenotype of cervical cancer cells [13]. To date, many studies have reported the technology of using siRNA to knockdown HPV16-E7 mRNA, thus restoring the expression of tumor suppressor pRb and induce apoptosis in HPV infected cells [14]. However, the effect of siRNA tends to be counteracted by human cells in the long run and cannot be inherited [15], narrowing its application. Therefore, in our study, we engineered E7-specific gRNAs/Cas9 to disrupt viral oncogene E7 at DNA level (Figure 1). The nuclease activities of the four customized gRNAs/Cas9 targeting E7 gene were first tested in the SSA system (Figure 2) and then confirmed by T7E1 assay (Figures 4(e) and 4(f)). When these four pairs of gRNA/Cas9 endonuclease system were introduced into both HPV16 positive and HPV16 negative cell lines, only HPV16 positive SiHa and Caski cell lines displayed high rate of apoptosis (Figure 3).

The induced apoptotic effect of RNA-guided Cas9 endonuclease may be attributed to NHEJ repairing mechanism upon repairing of DSBs in human cells, which will create point mutations or frameshift mutations of E7 gene [10], resulting in compromised E7 function. Consistent with this speculation, we did observe decreased protein expression of E7 gene and restoration of tumor suppressor protein pRB (Figures 4(g) and 4(h)). However, we could not rule out another possibility that the apoptotic effect of RNA-guided Cas9 endonuclease was resulted from the DSB event, instead of compromised function of viral oncogenes. For example, if DSBs were left unrepaired, they could directly lead to permanent cell cycle arrest, apoptosis, or mitotic cell death [16]. Much more work needs to be done to gain deeper understanding of Cas9-mediated effect. For instance, in CRISPR/Cas9 treated host cells, expression levels of E2F transcription factors should be investigated to check the downstream effects of E7 on cellular proliferation. And DNA cell cycle analysis using propidium iodide should also be performed to monitor the cell cycle stages. In addition, experiments on effects of gRNAs/Cas9 targeting different regions, for instance, long control region (LCR) of HPV genome, should be carried out in the future.

Another important concern of the CRISPR/Cas system as an antiviral agent is its specificity [17]. The recognition sequence 5′-N20NGG-3′ of the CRISPR/Cas system is relatively short and mismatches at its 5′-end are tolerable [18], making it prone to induce high off-target effects [17]. In our study, Cas9 induced apoptosis and cellular proliferation inhibition were observed only in HPV positive SiHa and Caski cells, but not in HPV negative C33A and HEK293 cells, suggesting, at least to some extent, that the CRISPR/Cas system was specific enough to distinguish HPV positive cells from HPV negative cells. Much more work still needs to be done to further improve the specificity of Cas9 system. For example, the paired-gRNA guided double nicking Cas9 system could be applied to extend the length of recognition sequence and reduce potential off-targets [19].

In summary, the results demonstrated for the first time that disrupting E7 gene using the CRISPR/Cas system can prompt cellular apoptosis, inhibit cellular proliferation, and restore expression of pRb protein in HPV16 positive cervical cancer SiHa and Caski cell lines. Therefore, targeting the HPV16-E7 gene using the CRISPR/Cas system might be a new therapeutic approach for the treatment of HPV infection and HPV-related cervical cancer.

## Figures and Tables

**Table 1:** Sequences of CRISPR gRNAs used in this paper.

Name	gRNA sequence (5′-3′)	PAM sequence (5′-3′)	DSB breaking site (bp) in HPV16 genome
gRNA-1	aacccagctgtaatcatgca	TGG	564
gRNA-2	acattgcatgaatatatgtt	CCT	583
gRNA-3	gagacaactgatctctactg	CCA	616
gRNA-4	gctggacaagcagaaccgga	CCA	688
HPV16E6-gRNA-1	gtcgatgtatgtcttgttgc	CCG	Not inducing DSB

**Table 2:** Sequences of primers used in this paper.

Name	Primers	Sequence (5′-3′)	Product size (bp)
gRNA-4-pair1	Forward	aacaaaccgttgtgtgatttg	501
	Reverse	taccatggctgatcctgcag	

gRNA-4-pair2	Forward	gtggaccggtcgatgtatgt	495
	Reverse	tgacgagaacgaaaatgacagt	
